# New Drugs, Appliances, and Things Medical

**Published:** 1890-08-30

**Authors:** 


### NEW DRUGS, APPLIANCES, AND THINGS MEDICAL.

[All preparations, appliances, novelties, etc., of which a notice is desired, should be sent for The Editor, to care of The Manager, 140, Strand, London, W.C.]

### KNIGHT'S ANTISEPTIC EUCALYPTUS TABLETS.

These are a combination of oil of eucalyptus with a large excess of carbonates. They are put up in various sizes, and have a paper of instructions with them. The oil appears to be of very good quality, and being in a state of fine division mixes fairly well with water. The idea is useful from its convenience, though we do not see that this method of using eucalyptus has any particular advantage over others before the public. We have so recently written at length on the value of eucalyptus as a disinfectant, that we need not now enlarge upon it. The tablets may be obtained from G. Voght and Co., 19, Laurence Pountney Lane, E.C.

